# Robust *H*_*∞*_ Control for Spacecraft Rendezvous with a Noncooperative Target

**DOI:** 10.1155/2013/579703

**Published:** 2013-08-20

**Authors:** Shu-Nan Wu, Wen-Ya Zhou, Shu-Jun Tan, Guo-Qiang Wu

**Affiliations:** ^1^State Key Laboratory of Structural Analysis for Industrial Equipment, Dalian University of Technology, Dalian 116024, China; ^2^School of Aeronautics and Astronautics, Dalian University of Technology, Dalian 116024, China

## Abstract

The robust *H*_*∞*_ control for spacecraft rendezvous with a noncooperative target is addressed in this paper. The relative motion of chaser and noncooperative target is firstly modeled as the uncertain system, which contains uncertain orbit parameter and mass. Then the *H*_*∞*_ performance and finite time performance are proposed, and a robust *H*_*∞*_ controller is developed to drive the chaser to rendezvous with the non-cooperative target in the presence of control input saturation, measurement error, and thrust error. The linear matrix inequality technology is used to derive the sufficient condition of the proposed controller. An illustrative example is finally provided to demonstrate the effectiveness of the controller.

## 1. Introduction

Recent decades have witnessed the prosperity and maturity of space technology, and the problem of spacecraft rendezvous has received detailed attention, as this is a key aspect for future missions which rely on the paradigms of spacecraft on-orbit service and space interception and capture [1–3]. Many control algorithms have been developed to perform rendezvous with a target spacecraft. According to different output modes of control force, the relative translation control of rendezvous maneuvers can be divided into impulse control and continuous thrust control [4]. The multi-impulse algorithm, which is an open-loop methodology, is studied to perform rendezvous [5–9]. With the development of control techniques and spacecraft thrusters, some novel closed-loop feedback algorithms are developed to achieve high precision and ideal robustness. Multiobjective control of spacecraft rendezvous is investigated in [10], and a robust state-feedback controller based on Lyapunov approach and liner matrix inequalities technique is proposed to deal with rendezvous problem in the presence of parametric uncertainties, external disturbances, and input constraints. The two-step sliding mode control to achieve the rendezvous problem with finite thrust in the presence of the Earth's gravitational perturbation is studied [11]. The robust orbital control problem for low earth orbit spacecraft rendezvous subjects to the parameter uncertainties, the constraints of small-thrust and guaranteed cost during the orbital transfer is studied in [12], and the controller design is cast into a convex optimization problem subject to linear matrix inequality (LMI) constraints. The robust *H*_*∞*_ control problem of spacecraft rendezvous on elliptical orbit is addressed in [13], and a sufficient condition for the existence of the robust *H*_*∞*_ controller is given in terms of the periodic Riccati differential equation. The model predictive control system to guide and control a chasing spacecraft during rendezvous with a passive target spacecraft in an elliptical or circular orbit is presented in [14]. A novel Lyapunov-based adaptive control strategy for spacecraft maneuvers using atmospheric differential drag is studied in [15], and the control forces required for rendezvous maneuvers at low Earth orbits can be generated by varying the aerodynamic drag affecting each spacecraft. The relative translation problem of spacecraft rendezvous is cast as a stabilization problem addressed using Lyapunov theory [16]. A new control scheme for relative translation of spacecraft formation flying, including the triple-impulse strategy for the in-plane motion, the single-impulse maneuver for the cross-track motion, and the time-optimal aerodynamic control for the along-track separation, is proposed in [17].

Although the abovementioned control algorithms have shown adequate reliability in relative translation control, they only focus on the rendezvous and proximity maneuvers with a cooperative target spacecraft. To the best knowledge of the authors, there are very few research works on the control problem of rendezvous with a noncooperative target. A Lyapunov min-max approach-based feedback control law is proposed to deal with the autonomous rendezvous problem with an escaped noncooperative target [18]. A fuzzy controller is developed to perform rendezvous with a noncooperative target considering uncertainties in orbital maneuver and attitude tumbling [19]. Based on the CW equations, a robust *H*-two/*H*-infinity controller is proposed to perform interception maneuvers for target satellite with parametric uncertainties, external disturbances, and control input constraint [20]. For rendezvous with a noncooperative target spacecraft, two critical problems should be addressed. On the one hand the orbit parameters of the noncooperative target cannot be determined precisely, which therefore make the relative translation as an uncertain system. These uncertainties have much to do with the stability and accuracy of rendezvous. On the other hand, it is generally required to achieve relative translation with less fuel consumption in finite time [21]. Then the synthesized problem of finite rendezvous time and fuel consumption, which can be defined as the finite time performance, should be addressed for rendezvous with a noncooperative target. Current works have not taken the both aspects into consideration simultaneously. In practice, the orbital control input force is limited, which can be divided into control input constraint and control input saturation. All of above issues make it difficult to achieve an ideal control performance for rendezvous with a noncooperative target.

To advance the control problem of relative translation of rendezvous with a noncooperative spacecraft, the robust *H*_*∞*_ control approach is developed in this paper. The relative motion of chaser and noncooperative target is modeled as the uncertain system. A robust *H*_*∞*_ controller is then designed to achieve rendezvous in the presence of control input saturation, measurement error, and thrust error, and the *H*_*∞*_ performance and finite time performance are guaranteed. An illustrative example is finally presented to demonstrate the performance of proposed controller.

## 2. Problem Definition

### 2.1. Relative Motion Dynamics

The orbit of the noncooperative target spacecraft is assumed to be circular, and then the motion of the chaser, relative to the target, can be governed by the following equations [4]:

(1) $$\begin{array}{c} \ddot{x}-2\omega \dot{y}-3\omega^{2}x=\frac{1}{m}T_{x},\\ \ddot{y}+2\omega \dot{x}=\frac{1}{m}T_{y},\\ \ddot{z}+\omega^{2}z=\frac{1}{m}T_{z}, \end{array}$$

where *x*, *y*, and *z* represent the relative position of chaser with reference to target, *ω* denotes the orbit angular velocity of the target moving around the Earth, *m* represents the mass of chaser spacecraft, and *T*_*x*_, *T*_*y*_, and *T*_*z*_ denote the control forces. Defining the state vector $X={\left[x,y,z,\overset{.}{x},\overset{.}{y},\overset{.}{z}\right]}^{T}$, output vector *Y* = [*x*,*y*,*z*]^*T*^, and the control input vector *u* = [*T*_*x*_,*T*_*y*_,*T*_*z*_]^*T*^, (1) can be rewritten as

(2) $$\begin{array}{c} \overset{.}{X}=AX+Bu,\\ Y=CX, \end{array}$$

where *Y* is the output vector:

(3) $$\begin{array}{c} A=\left[\begin{array}{cccccc} 0 & 0 & 0 & 1 & 0 & 0\\ 0 & 0 & 0 & 0 & 1 & 0\\ 0 & 0 & 0 & 0 & 0 & 1\\ 3\omega^{2} & 0 & 0 & 0 & 2\omega & 0\\ 0 & 0 & 0 & -2\omega & 0 & 0\\ 0 & 0 & -\omega^{2} & 0 & 0 & 0 \end{array}\right],\\ B=\frac{1}{m}{\left[\begin{array}{cccccc} 0 & 0 & 0 & 1 & 0 & 0\\ 0 & 0 & 0 & 0 & 1 & 0\\ 0 & 0 & 0 & 0 & 0 & 1 \end{array}\right]}^{T},\\ C={\left[\begin{array}{ccc} 1 & 0 & 0\\ 0 & 1 & 0\\ 0 & 0 & 1\\ 0 & 0 & 0\\ 0 & 0 & 0\\ 0 & 0 & 0 \end{array}\right]}^{T}. \end{array}$$

As rendezvous with a noncooperative target, the orbit angular velocity *ω* cannot be determined precisely. It can be then characterized as

(4) $$\begin{array}{c} \omega =\omega_{0}\left(1+\varphi_{1}\left(t\right)\right), \end{array}$$

where $\omega_{0}=\sqrt{\mu /r^{3}\;}$ represents nominal value, and *φ*_1_(*t*) represent the uncertain component. During the relative translation, the mass of chaser spacecraft could change and then is given by

(5) $$\begin{array}{c} m=m_{0}+\Delta m=m_{0}\left(1+\phi_{2}\left(t\right)\right), \end{array}$$

where *m*_0_ is the nominal value, Δ*m* represents the uncertain component of *m*, which arises from fuel consumption and payload variation, and *ϕ*_2_(*t*) = Δ*m*/*m*_0_. The uncertain components can be assumed that

(6) $$\begin{array}{c} \left|\phi_{1}\left(t\right)\right|\leq \delta_{1},\;\;\left|\phi_{2}\left(t\right)\right|\leq \delta_{2}. \end{array}$$

Combing (2)–(5), (2) can be rewritten as

(7) $$\begin{array}{c} \overset{.}{X}=\overset{-}{A}X+\overset{-}{B}u,\\ Y=CX, \end{array}$$

where

(8) A−=A0+ΔA,  B−=B0+ΔB,  ΔA=ΩEM,ΔB=ΩEN,E=diag⁡(E1,E2,…,E7),A0=[0001000000100000013ω020002ω00000−2ω00000−ω02000],B0=1m0[000000000100010001],  N=δ2δ2−1[000000000100010001],Ω=[0000000000000000000003ω02002ω0m0−10000−2ω000m0−100−ω020000m0−1],M=[2δ1+δ1200000002δ1+δ12000000δ1000000δ10000000000000000000],E1=E2=2φ1(t)+φ12(t)2δ1+δ12,  E3=E4=φ1(t)δ1,E5=E6=E7=φ2(t)(1−δ2)δ2(1+φ2(t)).

**Remark 1**

*Ω*, *M*, and *N* are real constant matrixes and *E* denotes an uncertain real matrix, which represents the uncertainties of system (7). *Ε* is defined as the norm-bounded uncertain parameter and satisfies

(9) $$\begin{array}{c} E^{T}E\leq I, \end{array}$$

where *I* is the identity matrix.

### 2.2. Notations, Definitions, and Lemmas

**Notation 1**

The notations used in the paper are presented. The superscript *T* stands for matrix transposition. For a symmetric matrix Ψ, the notation Ψ > 0  (Ψ < 0) denotes its positive (negative) definiteness. diag⁡(⋯) represents a block-diagonal matrix. In symmetric block matrices or complex matrix expressions, we use an asterisk (∗) to represent a term that is induced by symmetry,

**Definition 2 (*H*_*∞*_ performance)**

For such a continuous system:

(10) $$\begin{array}{c} \Phi :\overset{.}{z_{1}}=Q_{1}z_{1}+Q_{2}w,\\ z_{2}=Q_{0}z_{1}. \end{array}$$

Define the transfer function matrix from *w*(*t*) to *z*_2_(*t*)

(11) $$\begin{array}{c} \phi \left(s\right)=Q_{0}{\left(sI-Q_{1}\right)}^{-1}Q_{2}, \end{array}$$

where *I* is the unit matrix. The *H*_*∞*_ norm of *ϕ* is given by

(12) $$\begin{array}{c} {||\phi ||}_{\infty }=\underset{\omega }{\sup}\;\sigma_{\max}\left(\phi \left(j\omega \right)\right), \end{array}$$

where *σ*_max⁡_(·) denotes the maximum singular value, sup⁡ represents the supremum, ||·||_*∞*_ is the *H*_*∞*_ norm, and *ω* is the system frequency. The *H*_*∞*_ performance is governed by the following inequality:

(13) $$\begin{array}{c} {||\phi ||}_{\infty }<\Theta , \end{array}$$

where Θ denotes a positive constant. Under zero initial condition, (13) can be rewritten as [22]

(14) $$\begin{array}{c} {||z_{2}||}_{2}^{2}<\Theta^{2}{||w||}_{2}^{2}, \end{array}$$

where ||·||_2_ represents the *L*2 norm.

**Definition 3 (finite time performance)**

The system finite time performance is given by

(15) $$\begin{array}{c} J=x^{T}\left(t_{f}\right)R_{1}x\left(t_{f}\right)+\int_{t_{0}}^{t_{f}}\left(x^{T}\left(t\right)R_{2}x\left(t\right)+u^{T}R_{3}u\right)dt, \end{array}$$

where *x*(*t*) is system state, *R*_1_, *R*_2_, and *R*_3_ are positive diagonal matrixes, *u* represents control input, and *t*_0_ and *t*_*f*_ denote initial time and terminal time.

**Lemma 4**

Let *υ*_1_ and *υ*_2_ be the vectors of dimension *m*, Π is a matrix with same dimension *m* × *m*, and then the following inequality holds if  Π^*T*^Π ≤ *I* [23]:

(16) $$\begin{array}{c} 2\upsilon_{1}^{T}\Pi \upsilon_{2}\leq \upsilon_{1}^{T}\upsilon_{1}+\upsilon_{2}^{T}\upsilon_{2}. \end{array}$$

## 3. Controller Design

In this section, we will investigate the control problem of spacecraft rendezvous with a noncooperative target. The *H*_*∞*_ approach is employed to propose the following controller:

(17) $$\begin{array}{c} u=KX, \end{array}$$

where *K* is a constant feedback control gain to be determined. In practice, the measurement error and thrust error exist in an actual system, and stability robustness in presence of measurement errors and thrust errors is a primary consideration for design of any rendezvous control system [24]. Then (7) can be rewritten as

(18) $$\begin{array}{c} \overset{.}{X}=\overset{-}{A}X+\overset{-}{B}u+\overset{-}{B}d,\\ Y=CX, \end{array}$$

where *d* = *K*Δ*X* + Δ*u*, Δ*X*, and Δ*u* represent measurement error and thrust error, respectively.

In view of the limited power of actuator, the actual control input is generally limited. For a spacecraft rendezvous control system, thruster output has the saturation characteristic. Namely,

(19) $$\begin{array}{c} \overset{.}{X}=\overset{-}{A}X+\overset{-}{B}\tilde{u}+\overset{-}{B}d,\\ Y=CX, \end{array}$$

where $\tilde{u}=\text{Sat}(u)={\left[\text{Sat}(T_{x}),\text{Sat}(T_{y}),\text{Sat}(T_{z})\right]}^{T}$. The saturation function is given by

(20) $$\begin{array}{c} \text{Sat}\left(T_{i}\right)=\{\begin{array}{cc} -T_{\max}, & T_{i}<-T_{\max}<0\;\\ T_{i}, & -T_{\max}\leq T_{i}\leq T_{\max}\\ T_{\max}, & T_{i}>T_{\max}>0, \end{array} \end{array}$$

where *T*_max⁡_ is the maximum control force, and *i* = *x*, *y*, *z*. According to Definition 2, the *H*_*∞*_ performance of system (19) is given by [22]

(21) $$\begin{array}{c} {||Y||}_{2}^{2}<\gamma^{2}{||d||}_{2}^{2}, \end{array}$$

where *γ* is a positive constant. As the saturation characteristic, the finite time performance of system state and control input vector is governed by the following equation:

(22) $$\begin{array}{c} \Gamma =X^{T}\left(t_{f}\right)Q_{1}X\left(t_{f}\right)+\int_{t_{0}}^{t_{f}}\left(X^{T}\left(t\right)Q_{2}X\left(t\right)+{\tilde{u}}^{T}Q_{3}\tilde{u}\right)dt, \end{array}$$

where *Q*_1_, *Q*_2_, and *Q*_3_ denote positive diagonal matrixes. Γ represents the synthesized optimal value of rendezvous time and fuel consumption. Therefore, the control problem of spacecraft rendezvous with a noncooperative target is to determine the controller gain *K* such that *X* = 0 can be achieved and the *H*_*∞*_ performance of the system (19) is guaranteed subject to uncertainties, errors, and control input saturation, and the finite time performance Γ reaches the minimum value.

**Theorem 5**

For the uncertain rendezvous system (19) and a given scalar *γ* > 0, the closed-loop system is robustly asymptotically stable, the *H*_*∞*_ performance satisfies (21), and Γ has upper bound, if there exists a positive definite symmetric matrix *P* satisfying

(23) [PA^+A^TP+PB− B−TP+K^TK^+CTC+Q2+4K^TQ3K^PB−∗−γ2I]  <0,

(24) $$\begin{array}{c} Q_{1}-P<0, \end{array}$$

where $\hat{A}=\overset{-}{A}+\overset{-}{B}\hat{K}$, and $\hat{K}=0.5K$.

**Proof**

The proof includes two consecutive steps: (i) the system is robustly asymptotically stable, and (21) holds and (ii) Γ < *X* ^*T*^(*t*_0_)*PX*(*t*_0_).

Equation (19) can be rewritten as

(25) $$\begin{array}{c} \overset{.}{X}=\hat{A}X+\overset{-}{B}\tau +\overset{-}{B}d,\\ \tau =\text{Sat}\left(2\hat{K}X\left(t\right)\right)-\hat{K}, \end{array}$$

where *τ* satisfies

(26) $$\begin{array}{c} \tau^{T}\tau <X^{T}\left(t\right){\hat{K}}^{T}\hat{K}X\left(t\right). \end{array}$$

We define *P* as a positive definite symmetric matrix, which satisfies inequality (23) and (24), and then the candidate Lyapunov function is given by

(27) $$\begin{array}{c} V\left(t\right)=X^{T}\left(t\right)PX\left(t\right). \end{array}$$

Computing the first-order derivative of *V* yields

(28) V˙=2XTPX˙=2XTP(A^X+B−τ+B−d)=XTA^TPX+XTPA^X+2XTPB−τ+XTPB−d+dTB−TPX.

Employing Lemma 4 and inequality (26) yields

(29) 2XTPB−τ≤XTPB−B−TPX+τTτ<XT(PB−B−TP+K^TK^)X.

Substituting inequality (29) into (28) yields

(30) V˙<XT(A^TP+PA^+PB−B−TP+K^TK^)X+XTPB−d+dTB−TPX.

According to inequality (23), we have

(31) $$\begin{array}{c} {\hat{A}}_{}^{T}P+P\hat{A}+P\overset{-}{B}{\overset{-}{B}}^{T}P+{\hat{K}}^{T}\hat{K}<0. \end{array}$$

Then if *d* = 0, inequality (30) can be rewritten as

(32) $$\begin{array}{c} \dot{V}<X^{T}\left({\hat{A}}_{}^{T}P+P\hat{A}+P\overset{-}{B}{\overset{-}{B}}^{T}P+{\hat{K}}^{T}\hat{K}\right)X<0. \end{array}$$

Namely, the closed-loop system is asymptotically stable.

If *d* ≠ 0, we have

(33) ||Y||22−γ2||d||22=∫0∞(YTY−γ2dTd)dt=∫0∞(YTY−γ2dTd+V˙)dt+V(0)−V(∞)≤∫0∞(YTY−γ2dTd+V˙)dt=∫0∞Σdt,

where $\Sigma =Y^{T}Y-\gamma^{2}d^{T}d+\dot{V}$. The following inequality holds:

(34) Σ<Σ+XT(Q2+4K^TQ3K^)X<XTCTCX−γ2dTd+XTA^TPX+XTPA^X+dTB−TPX +XT(PB−B−TP+K^TK^+Q2+4K^TQ3K^)X+XTPB−d.

Namely

(35) YTY−γ2dTd+V˙ <[XTdT]T  ×[PA^+A^TP+PB−B−TP+K^TK^+CTC+Q2+4K^TQ3K^PB−∗−γ2I]  ×[Xd].

It can be then concluded that

(36) $$\begin{array}{c} {||Y||}_{2}^{2}-\gamma^{2}{||d||}_{2}^{2}<0. \end{array}$$

The *H*_*∞*_ performance of system (19) is guaranteed, and the closed-loop system is robustly asymptotically stable. The proof of Step 1 is completed.

The finite time performance Γ satisfies

(37) Γ<XT(tf)Q1X(tf)+∫t0tf(XT(t)Q2X(t)+uTQ3u)dt=XT(tf)Q1X(tf)+∫t0tfXT(Q2+4K^TQ3K^)Xdt.

By inequality (23), we obtain

(38) $$\begin{array}{c} P\hat{A}+{\hat{A}}^{T}P+P\overset{-}{B}{\overset{-}{B}}^{T}P+{\hat{K}}^{T}\hat{K}+Q_{2}+4{\hat{K}}^{T}Q_{3}\hat{K}. \end{array}$$

Employing inequalities (24) and (32), inequality (37) can be rewritten as

(39) Γ<XT(tf)Q1X(tf) −∫t0tfXT(PA^+A^TP+PB−B−TP+K^TK^)Xdt≤XT(tf)Q1X(tf)−∫t0tfV˙dt≤XT(tf)Q1X(tf) −∫t0tfddt(XT(t)PX(t))dt≤XT(tf)(Q1−P)X(tf)+XT(t0)PX(t0)≤XT(t0)PX(t0).

Hence Γ has upper bound *X* ^*T*^(*t*_0_)*PX*(*t*_0_). Generally, there exist *Q*_1_, *Q*_2_, and *Q*_3_ such that *X* ^*T*^(*t*_0_)*PX*(*t*_0_) is minimum. Combining Steps 1 and 2 then completes the proof of Theorem 5.

## 4. Illustrative Example

In this section, we provide an example to illustrate and validate the controller proposed above. The mass of chaser is *m*_0_ = 1000 kg, the target is moving along a circular orbit of radius *r* = 6420 km, and the gravitational constant is *μ* = 3.986 × 10^5^ km^3^/s^2^; thus, the normal orbit angular velocity is *ω*_0_ = 4.3633 × 10^−4^ rad/s. The upper bounds are *δ*_1_ = *δ*_2_ = 0.1, and *T*_max⁡_ = 400  *N*. The initial value is *X*(*t*_0_) = [2500,−2000,1200,−12,10,−5]^*T*^, Δ*X* = [0.05,0.05,0.05,0.01,0.01,0.01]^*T*^, and Δ*u* is less than 10% of control input *u*. The *H*_*∞*_ performance parameters are *γ* = 0.1. By using the LMI toolbox of MATLAB, we obtain the following associated matrices:

(40) K=[−2.7738−0.2650.2902−147.5603−15.638914.3656−0.401−2.6312−0.1378−15.6386−145.306−2.66030.2923−0.0731−2.761914.3644−2.6612−134.5208],Q1=diag⁡(2.007,…,2.007)6  ×  6  ×10−8,  Q2=diag⁡(1.2128,…,1.2128)6  ×  6  ×10−9,Q3=diag⁡(1.8244,1.8244,1.8244)  ×10−9.

**Figure 1:**
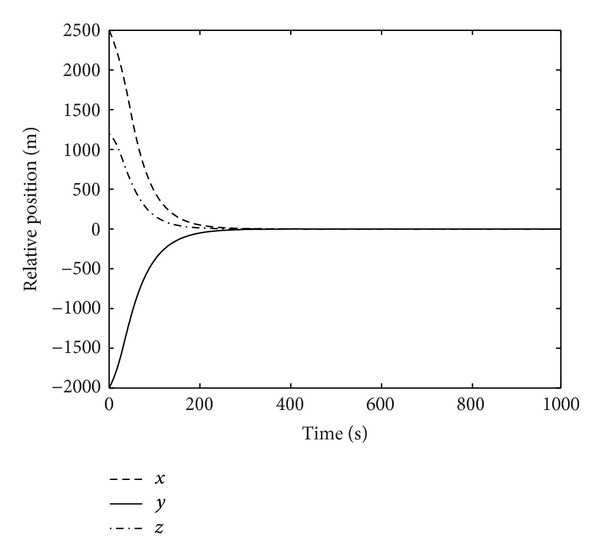
Relative position.

**Figure 2:**
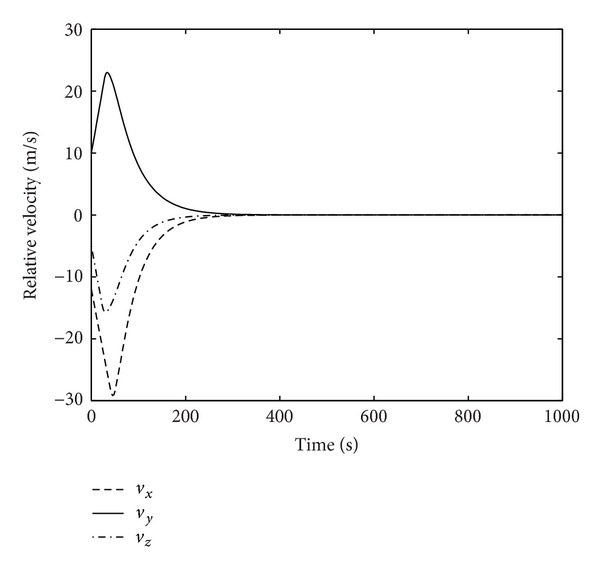
Relative velocity.

**Figure 3:**
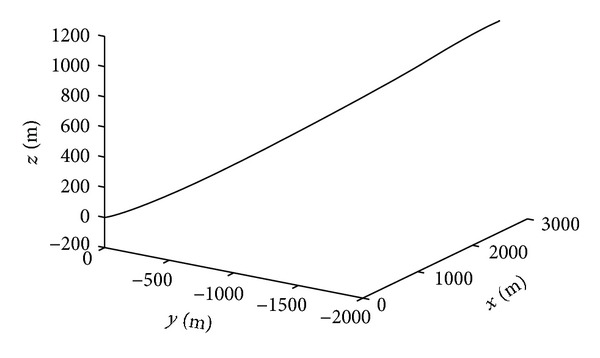
Rendezvous orbit.

**Figure 4:**
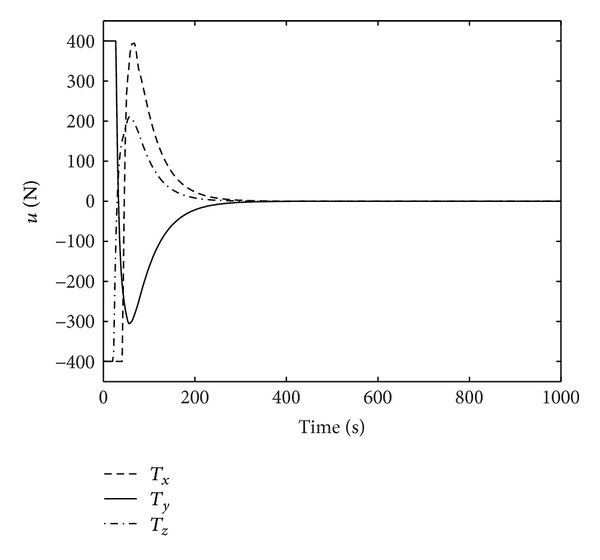
Control input.

Figures 1–4 show the simulation results of spacecraft rendezvous system. Figures 1 and 2 are the relative position and relative velocity of chaser and noncooperative target. As shown, the state vector *X* = 0 can be achieved after 300 seconds in the presence of uncertainties, errors, and control input saturation.  Figure 3 depicts the rendezvous orbit, and it can be seen that the chaser will eventually asymptotically rendezvous with the noncooperative target. The variation of control input force is presented in Figure 4. The control force reaches maximum value *T*_max⁡_ = 400  *N* in 50 seconds, which is due to the initial state, while the maximum control force of three axes is no large than *T*_max⁡_, which means that the control input saturation can be guaranteed by the proposed controller.

## 5. Conclusion

This paper has studied the robust *H*_*∞*_ control for spacecraft rendezvous with a noncooperative target subject to parameter uncertainty, finite time performance, and control input saturation. The relative motion of chaser and noncooperative target is modeled as an uncertain system. A robust *H*_*∞*_ controller, based on Lyapunov method and LMI techniques, is designed to drive the chaser to rendezvous with the noncooperative target. It should be noted that the finite time performance of closed-loop system is achieved. An illustrative example is finally presented to demonstrate that the proposed controller is robust to parameter uncertainties, measurement error, and control input saturation.
